# Inhibition of Carbonyl Reductase 1 Enhances Metastasis of Head and Neck Squamous Cell Carcinoma through β-catenin-Mediated Epithelial-Mesenchymal Transition

**DOI:** 10.7150/jca.34303

**Published:** 2020-01-01

**Authors:** Miyong Yun, Ae Jin Choi, Seon Rang Woo, Joo Kyung Noh, Ji-Youn Sung, Jung-Woo Lee, Young-Gyu Eun

**Affiliations:** 1 Department of Bioindustry and Bioresource Engineering, College of Life Sciences, Sejong University;; 2 Department of Otolaryngology-Head & Neck Surgery, School of Medicine, Kyung Hee University;; 3 Department of Biomedical Science and Technology, Graduate School, Kyung Hee University;; 4 Department of Pathology, School of Medicine, Kyung Hee University,; 5 Department of Oral and Maxillofacial Surgery, School of Dentistry, Kyung Hee University, Seoul, Republic of Korea.

**Keywords:** Carbonyl reductase, metastasis, head and neck squamous cell carcinoma, reactive oxygen species, epithelial-mesenchymal transition

## Abstract

**Objective:** Human carbonyl reductase 1 (CBR1) plays key roles in the regulation of oxidative stress and tumor progression. However, the detailed mechanism and clinical correlation between CBR1 and tumor progression in head and neck squamous cell carcinoma (HNSCC) is largely unexplored. This study will focus the effects of CBR1 on head and neck cancer progression and explore the possible mechanisms.

**Materials and Methods:** *CBR1* mRNA expression was analyzed according to lymph node metastasis (LNM) status in patients with HNSCC from publicly available databases. CBR1 protein levels were measured and compared in HNSCC patient tissues, with or without metastasis, using immunohistochemistry (IHC). The invasive ability of HNSCC with modulated CBR1 expression was assayed using an invasion assay. Expression levels of EMT marker proteins were analyzed using immunoblotting.

**Results:** HNSCC patients with LNM showed lower expression of *CBR1* than those without LNM. In addition, IHC in tissues indicated that patients with LNM had relatively lower levels of CBR1 in cancer tissue. Consistently, *in vitro* invasion assay, we found that *CBR1* inhibition using specific short interfering RNA treatment resulted in two- to three-fold increased invasion ability of HNSCC cell lines. Also, we proved that depletion of *CBR1* activated marker proteins participating in epithelial-mesenchymal transition (EMT) signaling. *CBR1* inhibition increased levels of intracellular reactive oxygen species (ROS) in HNSCC cells leading to upregulation of β-catenin, one of main transcription factors that induce EMT-related genes.

**Conclusion:** Our findings suggested that CBR1 plays an important role in metastasis of HNSCC tumors via regulation of ROS-mediated β-catenin activity, and that CBR1 may be marker for progression of HNSCC to metastasis.

## Introduction

Head and neck squamous cell carcinoma (HNSCC) constitutes about 4% of all cancers worldwide, is the sixth most common cancer, and has significant morbidity and mortality 1. Metastasis is the leading cause of morbidity in patients with a variety of solid tumors. HNSCC tends to metastasize to the regional lymph nodes through the lymphatic system rather than spreading hematogenously 2. More than 50% of patients with HNSCC have regional lymph node metastasis at the time of diagnosis 1. Regional lymph node metastasis is related to low survival rate and is the most important factor in determining appropriate staging and treatment plans 1, 3.

Human carbonyl reductase 1 (CBR1) is a well-documented reductase known as an NADPH‑dependent enzyme and is a member of the short-chain dehydrogenase/reductase superfamily 4. Intracellular CBR1 plays an important role in protecting cells from oxidative stress (through reactive oxygen species (ROS)) via inactivation of highly reactive lipid aldehydes, such as 4-oxonon-2-enal (ONE) and 4 hydroxynon-2-enal (HNE) 5. Another important role of CBR1 is to regulate tumor progression, including growth, proliferation, invasion, and metastasis. Several recent reports indicated that CBR1 expression is highly correlated with tumor metastasis in some tumors, including oral squamous cell carcinoma 6, ovarian cancer 7, and endometrial cancer 8, even though the results of some cases were contradictory.

Epithelial-mesenchymal transition (EMT), involved in variety of cellular processes, such as embryonic development, wound healing, and cancer development, was first defined as phenotypic transition of epithelial cells to mesenchymal cells by Elizabeth Hay in the early 1980s 9-11. EMT, as well as invasion, is a necessary preliminary event before metastasis during tumor progression. EMT can usually be distinguished by the changes in expression of epithelial markers, including claudins, E-cadherin, Crumbs3, and protein associated with lin seven 1 (PALS1); or mesenchymal markers, including N-cadherin, vimentin, α-smooth muscle actin (α-SMA), and fibroblast activation protein (FAP) 12. These EMT markers are tightly regulated by transcription factors such as Snail, Slug (both named for their Drosophila counterparts), twist family BHLH transcription factor 1 (Twist), zinc finger e-box binding homeobox 1 (ZEB1), zinc finger e-box binding homeobox 2 (ZEB2), nuclear factor kappa B (NF-kB), and β-catenin, which regulate cell mobility, proliferation, cytoskeleton, and extracellular matrix 13, 14. In particular, the translocation of β-catenin to the nucleus by Wnt and E-cadherin signaling is a necessary event to regulate the EMT process, leading to activation of the transcription of the genes encoding Snail, Twist, and matrix metalloproteinase 7 (MMP-7), as one of main transcription factors 13. In addition, β-catenin expression is affected by a variety of cellular signaling events, including ROS and hypoxia 15.

ROS have highly reactive properties that affect diverse cellular processes, such as cell proliferation, apoptosis, autophagy, migration, DNA damage, and inflammation, and act as second messengers 16, 17. In particular, ROS-mediated EMT activation was recently highlighted as an important mechanism for tumor malignancy. ROS are involved in the regulation of the actin cytoskeleton, extracellular matrix (ECM) remodeling, cell-cell junctions, and mobility, all of which affect the EMT ability of a cell. EMT induction by ROS-mediated β-catenin modification is initiated from phosphorylation of Y654 of β-catenin as a transcriptional co-factor 15. Another study indicated that low levels of ROS could increase the β-catenin stability, leading to induction of the expression of endogenous Wnt target genes 18.

Hence, it is unknown whether CBR1 affects ROS-mediated EMT leading to metastatic tumors in HNSCC; however, it is likely because CBR1 is a key regulator of oxidative molecules. We hypothesized that inhibition of CBR1 would increase ROS levels, resulting in accumulation of β-catenin and concomitant upregulation of EMT genes *in vitro* and *in vivo*.

## Methods

### Patient dataset and gene expression data

All clinical and gene expression data are available from the National Center for Biotechnology Information Gene Expression Omnibus database (http://www.ncbi.nlm.nih.gov/geo). Gene expression data from the Institute for Medical Informatics, Statistics and Epidemiology (the Leipzig cohort, GSE65858, n = 270) and Vanderbilt University (the Vanderbilt cohort, GSE3292, n = 36) were used for analysis. The Leipzig cohort and Vanderbilt cohort were generated using an Illumina HumanHT-12 V4.0 expression bead chip and an Affymetrix Human Genome U133 Plus 2.0 Array, respectively 19.

### HNSCC tissue samples and immunohistochemistry

The formalin-fixed and paraffin-embedded HNSCC tissues (n = 42), with or without metastasis, were used in this study. The study was approved by the institutional review board (IRB) of Kyung Hee University Medical Center (KMC IRB- 2018-05-021). Immunohistochemistry (IHC) was carried out on 4-µm tissue sections using the Bond Polymer Refine Detection System 19.

### Immunohistochemistry interpretation and analysis

All sections were examined by an expert pathologist who was blinded to the clinical data. Detail procedures are exactly same with previous study 19.

### Cell culture

The HNSCC cell lines YD8 (Dr. Ahn, Kyung Hee Uiversity), SNU-1041 (Aju University), and YD10B, were purchased from Korean cell line bank (Seoul, Korea). All the cell lines were tested for the presence of *Mycoplasma* on December 11, 2017. YD8, SNU-1041, and YD10B were cultured in Roswell Park Memorial Institute (RPMI)-1640 medium (Corning, Manassas, VA, USA), supplemented with 10% fetal bovine serum (FBS; Corning) and 1% penicillin‑streptomycin (Corning). All the cell lines were cultured at 37 °C in the presence of 5% CO_2_.

### Short interfering RNA Transfection

HNSCC cells were plated at 60% confluency in a 60-mm dish 24 hours before transfection. Short interfering RNAs (siRNAs) were designed against specific target sequence of the human *CBR1* mRNA. The siRNAs were purchased from IDT (Cambridge, MA, USA). Scrambled duplex RNA was used as the control. The siRNA transfection was conducted using the TransIT-TKO Transfection Reagent (Mirus Bio, Madison, WI, USA) according to the manufacturer's recommendations.

### Overexpression cell line

GFP-conjugated empty or CBR1 plasmid have been previously described 19. HNSCC cells were plated at 60% confluency in a 60-mm dish 24 hours before transfection. The cells were transfected with plasmids using the TransIT-TKO Transfection Reagent (Mirus Bio, Madison, WI, USA) according to the manufacturer's recommendations.

### Western blotting

After transfection, cells were rinsed with ice-cold phosphate-buffered saline (PBS) and harvested using a cell scraper, followed by centrifugation. The cell pellets were lysed in RIPA buffer (50 mM Tris-HCl, 150 mM NaCl, 2 mM EDTA, and 1% TritonX-100) for 10 minutes on ice. After protein quantification (Micro-BCA Protein Assay, Pierce, Meridian, RD, USA), equal amounts of protein plus loading dye were added to lanes of an 8-15% sodium dodecylsulfate (SDS)-polyacrylamide gel, electrophoresed, and transferred to polyvinylidene difluoride membranes (Millipore, Billerica, MA, USA). The membranes were blocked and probed with primary antibodies recognizing CBR1 (Novurs, Littleton, CO, USA), E-cadherin, Vimentin, Slug, β-catenin, and β-actin (all from Cell Signaling, Beverly, MA, USA), and were then incubated with horseradish peroxidase-conjugated secondary antibodies (Cell Signaling). The protein‑antibody complexes were detected using enhanced chemiluminescence (GE healthcare, Little Chalfont, UK), according to the manufacturer's recommended protocol.

### Detection of ROS production

HNSCC cells were transfected with control siRNAs or gene-specific siRNAs for 40 hours. The level of intracellular ROS was then monitored using a total ROS detection kit according to the manufacturer's instructions (Enzo life sciences, Farmingdale, NY, USA). The cells were harvested, placed into 5-ml round-bottom polystyrene tubes after treatment, and washed with 1× wash buffer. The cells were centrifuged for 5 min at 400 ×* g* at room temperature and the supernatant was discarded. The cells were resuspended in 500 µl of ROS detection solution, stained at 37 °C in the dark for 30 min, and then analyzed by flow cytometry (BD Pharmingen, San Jose. CA, USA).

### Invasion assay

Transwell membranes (24-well, Costar, Cambridge, MA, USA) were coated with Matrigel (Corning) for 6 hours for the invasion assays. Cells (5 × 10^4^) in serum-free medium were seeded into each upper chamber, and 600 μl of medium supplemented with 10% FBS were added to each lower chamber. After incubation for 48 hours, cells adhering to the upper surface of the membrane were removed using a cotton swab. Cells that had invaded and were adhered to the lower surface were stained with 0.1% crystal violet and counted in four representative fields under light microscopy (200 × magnification).

## Results

### Patients with HNSCC with lymph node metastasis show comparatively lower expression of CBR1 compared to patients without lymph node metastasis

**Figure 1:**
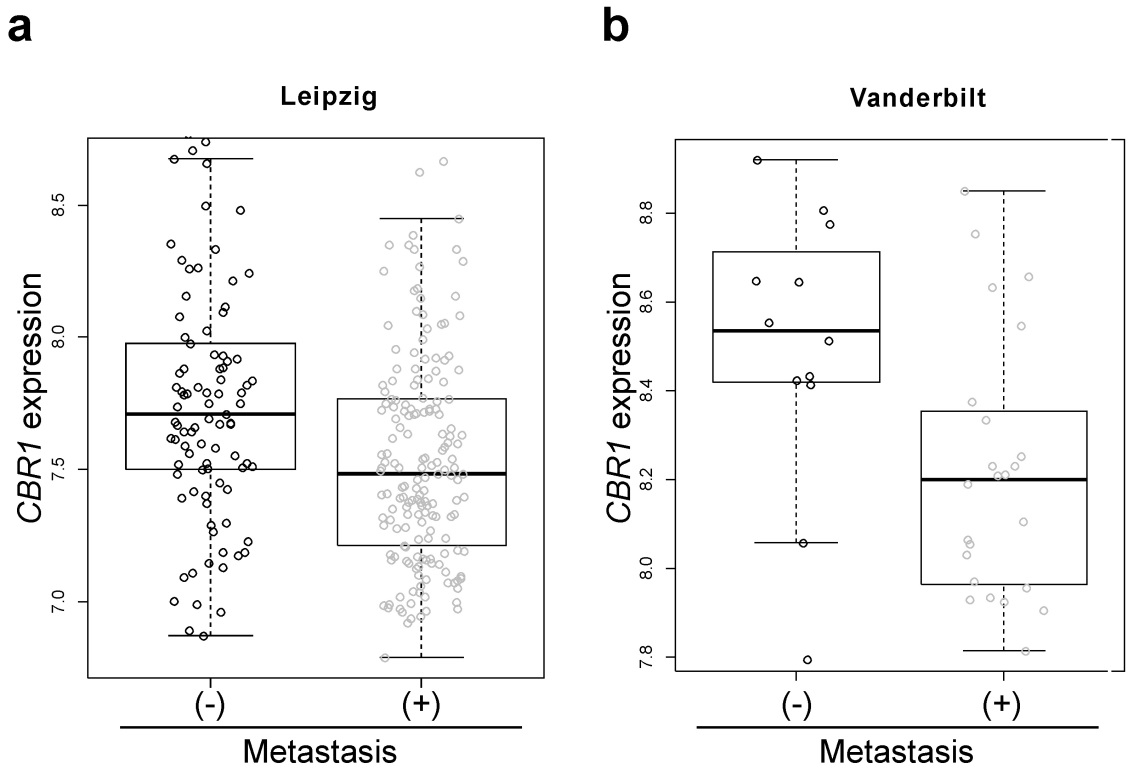
** Box-and-whisker plot of *CBR1* mRNA expression in patients with HNSCC with or without LNM.** a and b, The *CBR1* mRNA expression of patients with HNSCC with or without LNM in the Leipzig cohort (a, GSE65858, n = 270, with metastasis; n=177, without; n=94, p=9.53E-05) and Vanderbilt cohort (b, GSE3292, n = 36, with metastasis; n=12, without; n=24, p=0.027) from publicly available data were analyzed using the R program. (-): without metastasis, (+): with metastasis.

**Figure 2:**
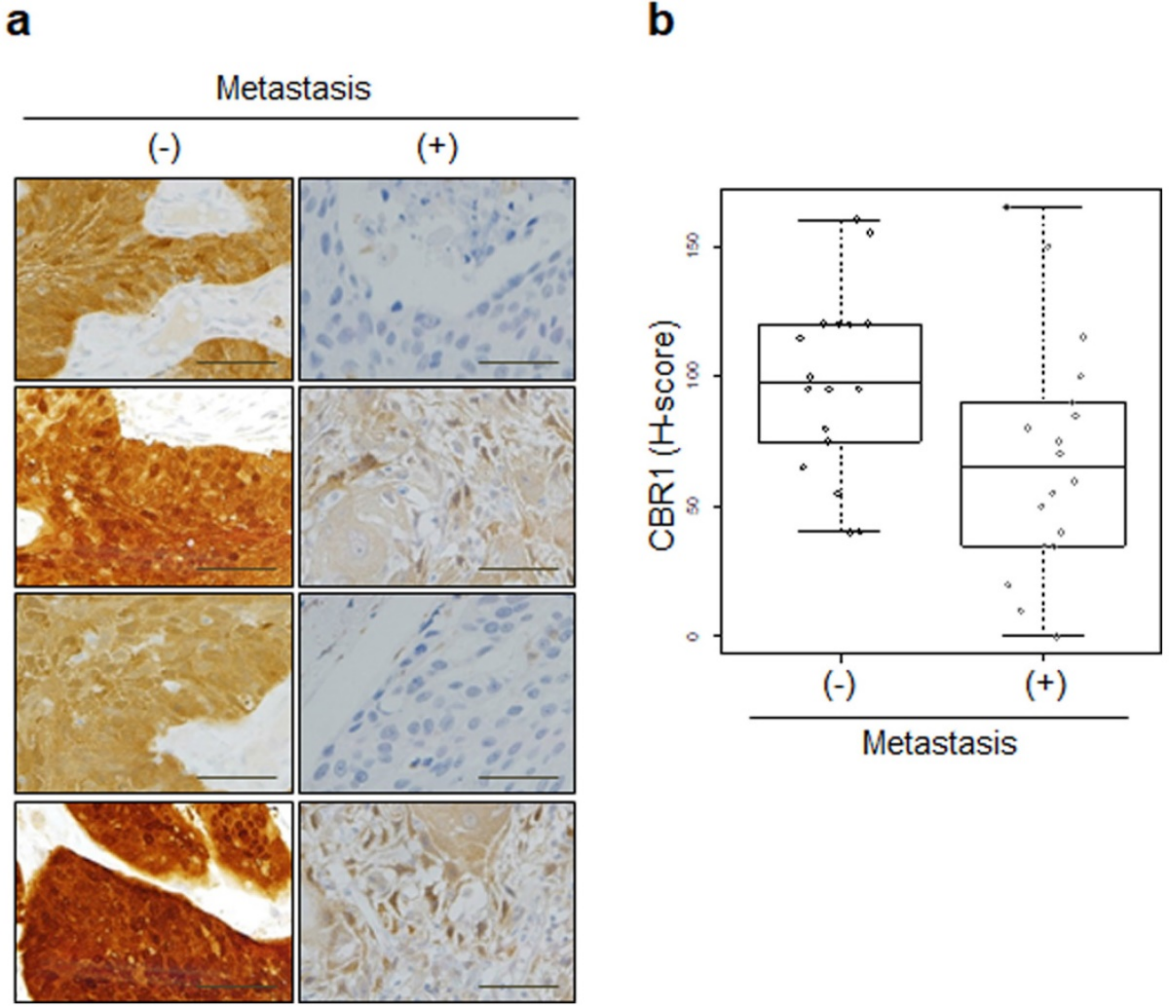
** CBR1 protein expression is highly correlated with LNM of HNSCC.** a, Immunohistochemistry showing the expression of CBR1 was conducted for cancer tissue from 36 patients with HNSCC (Kyung Hee Medical Center, with metastasis; n=18, without; n=18). An immunohistochemical staining score (H-score) was calculated by multiplying the intensity score and the fraction score (percentage of counted samples at each scale; see Materials and methods). b, Statistical analysis for the results shown in Fig. 2A results. Statistical analysis was performed using the R program. A Box-and-Whisker plot was used to compare the distribution of each sample in the groups (p=0.027). (-): without metastasis, (+): with metastasis.

To test the association of CBR1 with lymph node metastasis (LNM) in HNSCC, we compared the gene expression of CBR1 according to LNM status in HNSCC patient cohorts from publicly available data. The patients with LNM showed lower expression of* CBR1* in both the Leipzig and Vanderbilt cohorts (n=270, 7.76 ± 0.47 *vs*. 7.54 ± 0.42, p = 9.53E-05 and 8.50 ± 0.32 *vs*. n= 36, 8.22 ± 0.29, p = 0.0047, respectively) (Table 1, Fig. 1a and b). For validation, we compared the expression of CBR1 protein in cancer tissue of 36 patients with HNSCC using IHC (Table 1). Samples from patients with LNM had lower IHC scores than patients without LNM (106.9 ± 54.1 *vs*. 68.6 ± 44.8, p = 0.027) (Fig. 2a and b).

### Inhibition of *CBR1* increases the invasion ability of HNSCC cells

**Figure 3:**
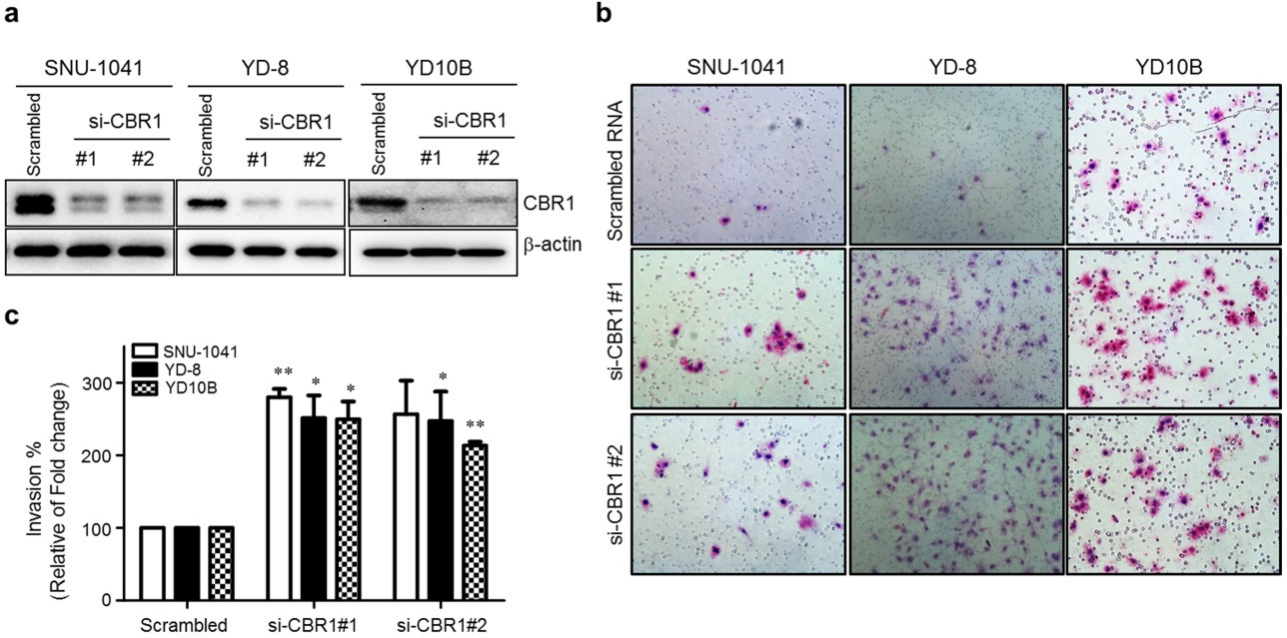
** Suppression of CBR1 increases the invasion ability of HNSCC cells.** HNSCC cells were transfected with scrambled or *CBR1*-specific siRNA. They were then plated onto the top of Matrigel-coated transwell inserts. Cells that had invaded the Matrigel were counted 48 h later. Invaded cells on the underside of the transwell filters were stained with crystal violet solution and imaged. a. The CBR1 protein level was monitored in HNSCC cells transfected with scrambled or *CBR1* specific siRNA. At 24 h after siRNA transfection, cell pellets were collected for western blotting analysis. β-actin was used as an internal loading control. b. Representative images of the cell invasion ability assay are shown. c. Effects of different treatments on the invasion ability of HNSCC cells were determined by counting the total numbers of invading cells. Data are means ± SEM. (n = 3). *P < 0.05; **P < 0.01 *vs*. scrambled RNA. SEM, standard error of the mean.

Previous clinical data clearly indicated that the expression level of CBR1 is correlated highly with metastasis of HNSCC. To explore whether CBR1 participates in tumor cell proliferation, we downregulated *CBR1* expression in HNSCC cells by transfection with specific siRNAs (Fig. 3a). Supplementary Figure 1a and b show that *CBR1* depletion did not affect proliferation of HNSCC cells at any time point. In the invasion assay, we found that *CBR1* inhibition resulted in a two- to three-fold increase of invasive HNSCC cells (SNU-1041, YD-8, and YD10B), compared with that of the cells transfected with the scrambled siRNA (Fig. 3b and c). These results suggest that CBR1 increased the invasion ability of HNSCC cells but did not affect proliferation.

### CBR1 depletion upregulates EMT markers

**Figure 4:**
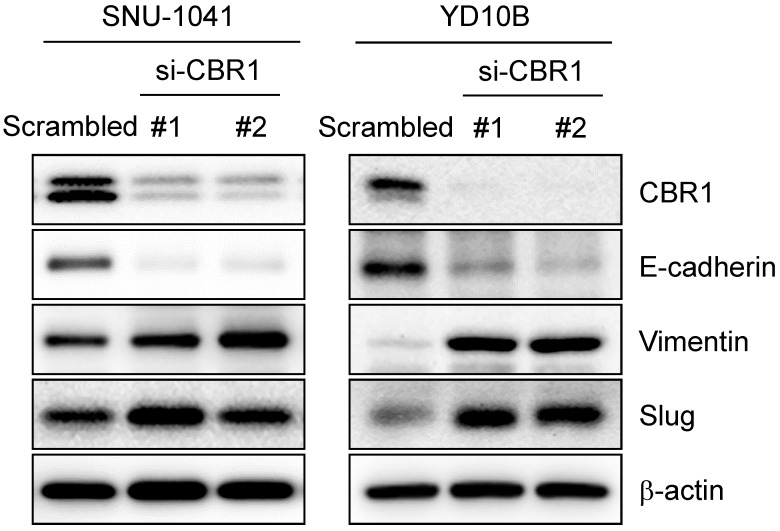
** Depletion of CBR1 upregulates EMT markers.** Western blotting analysis of the protein expression of EMT markers in HNSCC cells after *CBR1* siRNA transfection for 48 h. Western blotting analysis showed decreased E-cadherin levels and increased Vimentin and Slug levels in response to by CBR1 depletion. β-actin was used as an internal loading control.

EMT can usually be detected by the change in expression of epithelial or mesenchymal markers. EMT is a required first step for cancer cell invasion. To confirm whether inhibition of CBR1 expression affects EMT, we suppressed *CBR1* expression using the siRNA. CBR1 depletion significantly decreased the levels of the epithelial marker, E-cadherin, and increased mesenchymal markers, vimentin, and Slug compared with those in the control in HNSCC cells, SNU-1041 and YD10B (Fig. 4). These data indicate that CBR1 expression affects EMT regulation by modulating the levels of key proteins in the pathway.

### Depletion of CBR1 accumulates ROS increasing β-catenin levels in HNSCC cells

**Figure 5:**
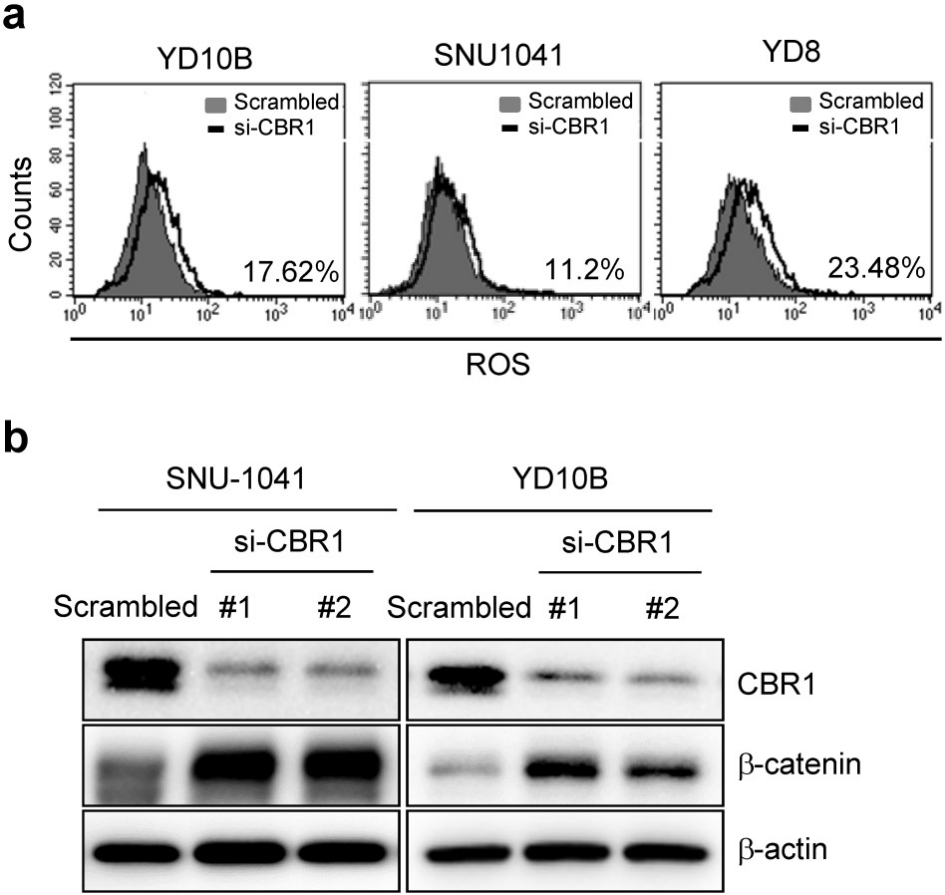
** Depletion of CBR1 accumulates ROS, leading to upregulation of β-catenin in HNSCC cells.** a, Scrambled RNA and* CBR1* siRNA were transfected into HNSCC cells, and 40 h later, ROS formation was analysed by FACS using a total ROS detection kit (Enzo). b, β-catenin and CBR1 protein level was monitored in HNSCC cells transfected with scrambled or *CBR1* specific siRNA. FACS, fluorescence activated cell sorting.

A previous study reported that downregulation of CBR1 increased vascular endothelial growth factor (VEGF)-C expression in ovarian cancer cells 7. To determine the molecular mechanism of CBR1-mediated invasion and/or metastasis, we first confirmed the expression of vascular endothelial growth factor (VEGF) genes in HNSCC cells (SNU-1041 and YD8). Except at 48 h in SNU-1041 cells, which showed a slight increase in *VEGFC* and *VEGFD* expression in response to siRNA #3, *CBR1* depletion using specific siRNAs did not affect the mRNA expression of the VEGF genes (*VEGFA*, *VEGFB*, *VEGFC*, and *VEGFD*) at 24 or 48 hours (Supplementary Fig. 2a and b). Next, we evaluated ROS levels and their downstream protein, β-catenin, in HNSCC cells after treatment with siRNAs against *CBR1*.* CBR1* inhibition increased the intracellular ROS level compared with that of YD10B, SNU1041 and YD8 cells transfected with the control siRNA (Fig. 5a). Expression of β-catenin, one of main transcription factors for EMT, is regulated by the ROS level in cancer cells. Therefore, we evaluated β-catenin expression in SNU-1041 and YD10B cells. Downregulation of *CBR1* expression significantly increased β-catenin levels in these cells (Fig. 5b). And the increased β-catenin protein was accumulated in both nucleus and cytoplasm (Supplementary Fig. 3a and b).

### Overexpression of CBR1 suppresses invasion and EMT of HNSCC cells

**Figure 6:**
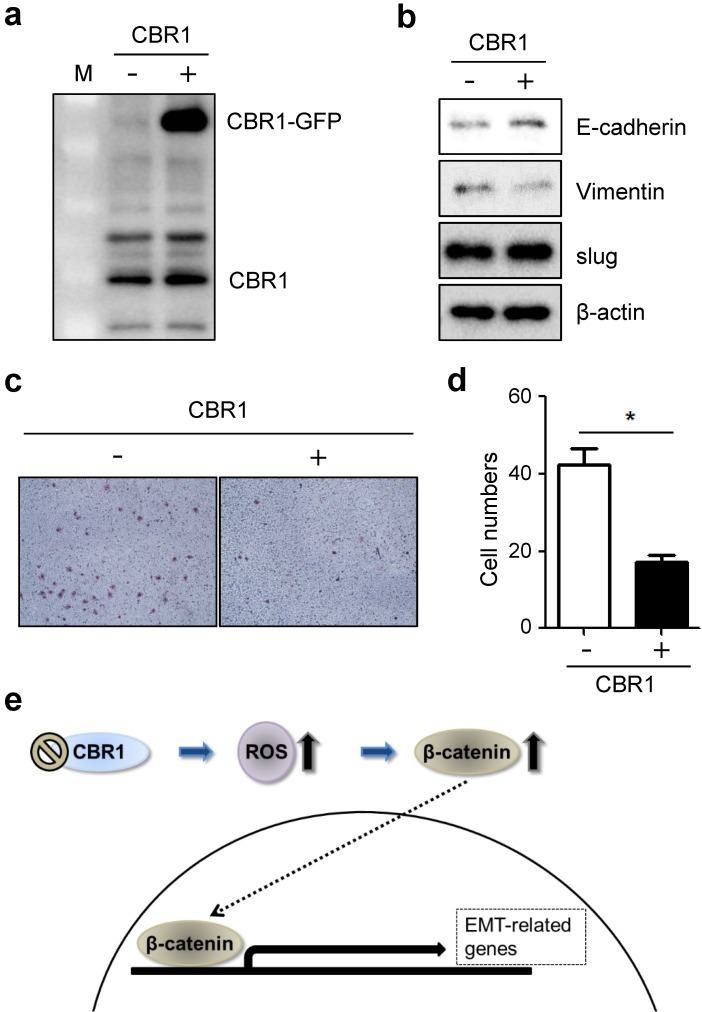
** Overexpression of CBR1 suppresses invasion and EMT of HNSCC cells.** YD10B cells were transfected with GFP-conjugated empty and CBR1 vectors. a, CBR1 protein expression was evaluated through western blotting analysis in transfected cells. b, Protein level of EMT markers were evaluated in the same samples under each conditions. c, Cells were characterized the invasiveness of empty and CBR1 vectors through invasion assay analysis using matrigel-coated transwells. d, Effects of different treatments on the invasion ability of YD10B cells were determined by counting the total numbers of invading cells. Data are means ± SEM. (n = 3). *P < 0.05 *vs*. empty vector. SEM, standard error of the mean. e, Scheme of CBR1 signaling pathway to regulate EMT in HNSCC cells.

Next, to confirm whether high expression level of CBR1 affects invasion and EMT of HNSCC cells, we constructed the cells overexpressing CBR1 (YD10B_CBR1) (Fig. 6a). CBR1 overexpression downregulates mesenchymal markers, vimentin, and upregulated the epithelial marker, E-cadherin compared with the control in HNSCC cells (Fig. 6b). In addition, invasive ability of cells overexpressing CBR1 (YD10B_CBR1) was significantly decreased compared with control cells (Fig. 6c and d). These data suggest that CBR1 expression may participate in regulation of EMT and invasion by modulating the levels of key proteins in the pathway.

## Discussion

The involvement of regional lymph nodes in patients with HNSCC is one of the most important prognostic factors. The presence of regional LNM also influences the choice of the adjuvant therapy used to inhibit disease recurrence 20. Patients with HNSCC with distant metastasis are often offered palliative care, because of limited treatment options. Therefore, in terms of clinical treatment and prediction, informative markers for metastatic processes, including EMT and invasion, of HNSCC are urgently required. In the present study, we sought to assess whether CBR1 expression correlates with metastasis and whether CBR1 inhibition affects invasion in patients with HNSCC and in HNSCC cell lines. The results clearly indicated that CBR1 expression correlates highly with metastasis in patients with HNSCC. In addition, downregulation of CBR1 expression affected the invasion and EMT of various HNSCC cells. These results suggested that CBR1 might be an effective target to treat metastatic HNSCC.

Despite advances in various imaging modalities, our ability to predict LNM and distant metastasis is limited. Therefore, the use of reliable marker proteins, including receptor tyrosine kinases (RTKs), signal transducer and activator of transcription 3 (STAT3), and NF-κB, have been considered an important way to increase precision of metastasis prediction in HNSCC 21. In addition, another emerging factor for metastasis prediction is Wnt/ β-catenin signaling, which participates in EMT regulation. Wnt signaling and/or β-catenin itself have a critical role in the initiation and progression of EMT in a variety of tumors, including breast cancer, hepatocellular carcinoma, and prostate cancer 22-24. Wnt ligands can induce transcriptional signal pathways, i.e., the so called the canonical (Wnt/β-catenin dependent) and noncanonical (β-catenin-independent) pathways 25, 26. The Wnt/β-catenin-dependent canonical pathway transcriptionally activates various genes participating in cell proliferation, development, cell division, stem cell renewal, and EMT 27-30. Accumulated and translocated β-catenin transcriptionally activates *MYC* (c-myc*)*, *CDKN1A* (cyclin dependent kinase Inhibitor 1A), and *CCND1* (cyclin D1), which can promote cell proliferation in pancreatic and hepatic cells, together with CCNE1 (cyclin E1) 25, 27. In contrast, based on our data, CBR1 inhibition‑mediated β-catenin accumulation did not affect cell proliferation, but only the expression of EMT-related genes. These data suggested that β-catenin, under conditions of depleted CBR1, might work with other specific transcriptional co-factors, which should be investigated further.

Accumulation of β-catenin, as well as its translocation to the nucleus, is a necessary event for Wnt signal transduction. The interaction between Wnt and the Frizzled-Axin -LRP-5/6 complex inhibits cytosolic GSK3B (glycogen synthase kinase 3 beta) leading to inhibition of β-catenin phosphorylation. Un-phosphorylated β-catenin can escape from the ubiquitin‑proteasome degradation machinery, causing it to accumulate in the cytoplasm 25, 31, 32. Although there are contradictory reports, ROS is another possible regulator of β‑catenin stability. Coant et al. recently reported that Nox-derived ROS partially regulates Notch and Wnt/β-catenin in the colon 33, 34. Another study confirmed that the induction of catalase, an antioxidant enzyme that degrades H_2_O_2_, suppressed Notch signaling pathways 35. Consistently, our data suggested that ROS is a major factor in the accumulation of β‑catenin in HNSCC in the context of suppressed CBR1 expression. However, the mechanisms by which CBR1‑mediated ROS increases β-catenin stability, and which factors participate in this process, remain to be determined.

Many types of tumor produce high levels of ROS via abnormal processes, including genetic, metabolic, and microenvironment-related alterations 36. These abundant ROS can affect tumor phenotypes, such as therapeutic resistance and metastasis. Therefore, it has been considered that uncontrolled redox balance and signaling is a common hallmark of tumors, leading to poor prognosis and high mortality. However, paradoxically, antioxidant systems that prevent ROS development in tumors are present. Therefore, proteins, for example superoxide dismutase (SOD) and glutathione peroxidase (GPX), which participate in the antioxidant response, have been studied for development as prognostic markers 37, 38. CBR1 is a well-known anti-oxidant enzyme that regulates intracellular ROS as an NADPH-dependent oxidoreductase 39. Previous reports suggested that CBR1 expression is related to tumor progression, such as in uterine endometrial cancer and uterine cervical squamous carcinoma 8. In addition, Osawa et al. proved that suppression of CBR1 expression is related to lymph node metastasis, leading to poor prognosis in ovarian cancer 7.

## Conclusions

Consistent with previous reports, our data showed that CBR1 inhibition enhanced the invasion and EMT capabilities via regulation of β-catenin signaling of HNSCC cells (Fig. 6e). A low level of CBR1 expression correlated with metastasis in patients with HNSCC. These data suggested that CBR1 could be a useful marker for HNSCC prognosis.

## Supplementary Material

Supplementary figures and tables.

## Figures and Tables

**Table 1:** Clinicopathological data of 36 patients with HNSCC

	Metastasis (-) Patients n (%)	Metastasis (+) Patients n (%)
Number	18	18
Age, mean (range)	62.4 (31-80)	61.6 (52 - 77)
Gender		
male	12 (66.7)	14 (77.8)
female	6 (33.3)	4 (22.2)
pT stage		
I	5 (27.8)	4(22.2)
II	7 (38.9)	5(27.8)
III	3 (16.7)	2(11.1)
IV	3 (16.7)	7(38.9)
pN stage		
0	18	0
I	0	4
II	0	14
III	0	0
pM stage		
0	18	18
I	0	0
Primary site		
Oral cavity	10 (55.6)	10 (55.6)
Oropharynx	3 (16.7)	4 (22.2)
Larynx	5 (27.8)	0 (0)
Hypopharynx	0 (0)	4 (22.2)

## Abbreviations

CBR1: carbonyl reductase 1
HNSCC: head and neck squamous cell carcinoma
LNM: lymph node metastasis
IHC: immunohistochemistry
EMT: epithelial-mesenchymal transition
ROS: reactive oxygen species
ONE: 4-oxonon-2-enal
HNE: 4-hydroxynon-2-enal
PALS1: protein associated with lin seven 1
α-SMA: α-smooth muscle actin
FAP: fibroblast activation protein
Twist: twist family BHLH transcription factor 1
ZEB1: zinc finger e-box binding homeobox 1
NF-kB: nuclear factor kappa B
MMP-7: matrix metalloproteinase 7
ECM: extracellular matrix
VEGF: vascular endothelial growth factor
STAT3: signal transducer and activator of transcription 3
SOD: superoxide dismutase
GPX: glutathione peroxidase
