# Supplementary material for: Inhibition of Carbonyl Reductase 1 Enhances Metastasis of Head and Neck Squamous Cell Carcinoma through β-catenin-Mediated Epithelial-Mesenchymal Transition
Source: J Cancer. 2020 Jan 1;11(3):533–41. doi: 10.7150/jca.34303 (PMC6959035; doi:10.7150/jca.34303)
Supplement: Supplementary file 1 — Supplementary figures and tables. [file jcav11p0533s1.pdf]

Supplementary figures and figure legends

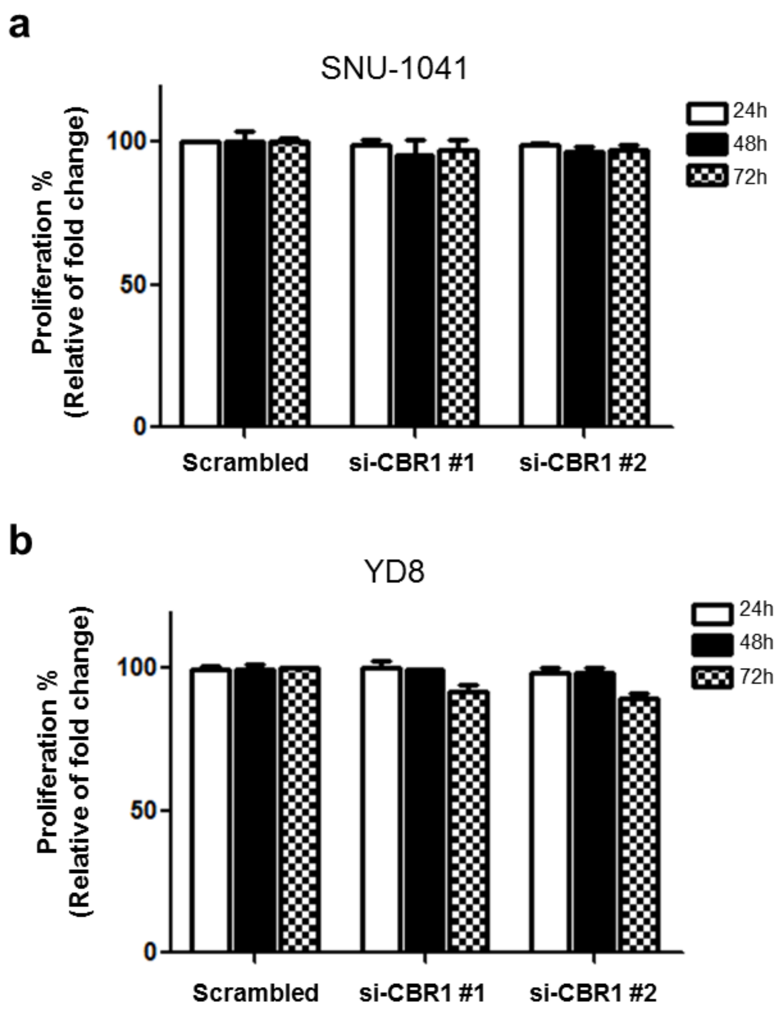

Supplementary Figure 1. CBR1 depletion does not effect on proliferation of HNSCC cells. SNU-1041(a) and YD8(b) were transfected with scrambled or *CBR1*-specific siRNA. They were then incubated for indicated time. After incubation, proliferation was assessed using a Cell Counting kit-8. Proliferation rate of CBR1 siRNA-transfected cells were compared with scramble siRNA and presented %. Data are presented as the mean ± SD.

a

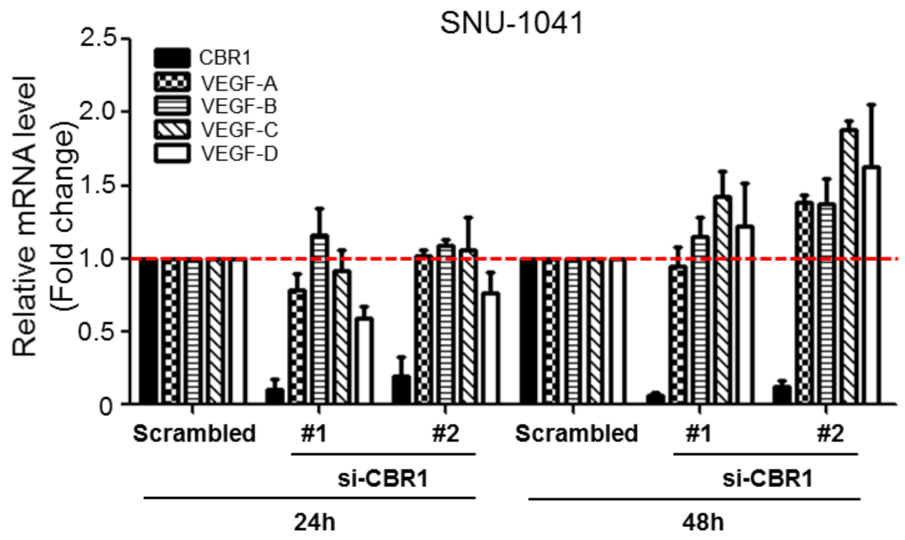

b

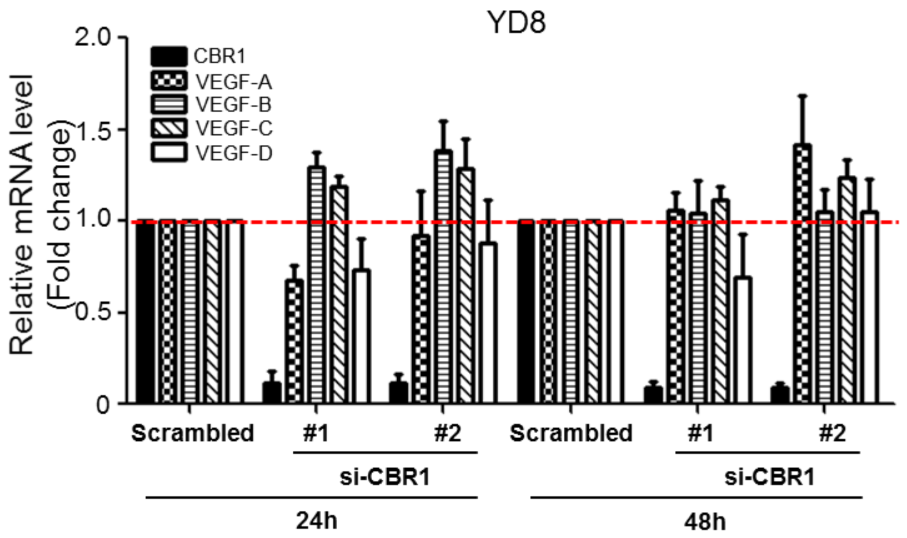

Supplementary Figure 2. CBR1 depletion is not highly correlated with VEGFs secretion in HNSCC cells. SNU-1041(a) and YD8(b) were transfected with scrambled or *CBR1*-specific siRNA. At 24 h after siRNA transfection, total RNA was extracted from the indicated cell lines and mRNA expression of VEGF subfamily genes were analyzed by RT-qPCR. GAPDH was used as an internal control to normalize the expression level of each gene. Data are presented as the mean ± SD.

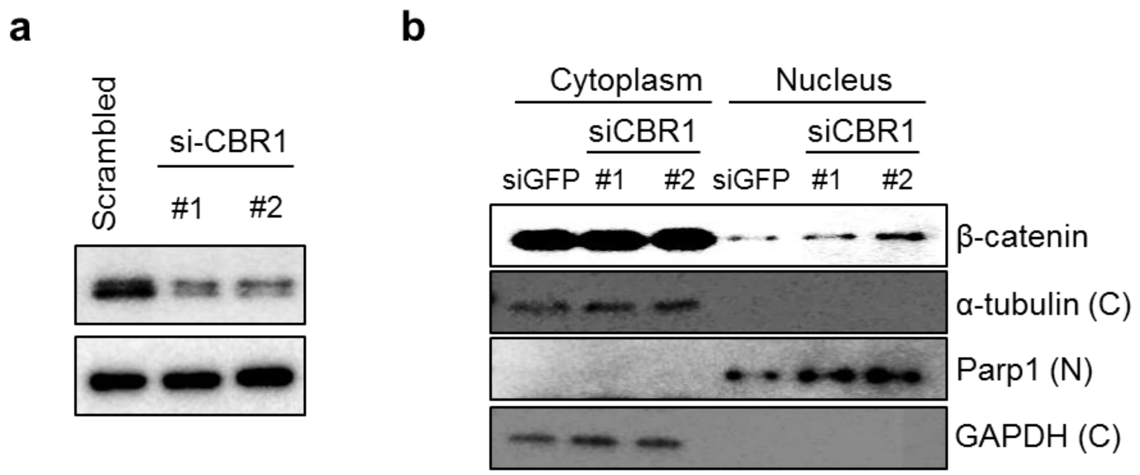

Supplementary Fig. 3. β-catenin under CBR1 depletion conditions accumulated more in cytoplasm than nuclear. SNU1041 cells were transfected with scrambled or *CBR1*-specific siRNA for 48 h. a, The CBR1 protein level was evaluated through western blotting analysis in the transfected cells. β-actin was used as an internal loading control. b, subcellular localization of β-catenin was analyzed by immunoblotting after nuclear/cytosol fractionation. α –tubulin, GAPDH and Parp1 were used as cytoplasmic and nuclear markers, respectively.

Supplementary materials and methods

Cell proliferation assay

Proliferation was assessed using a Cell Counting kit-8 (CCK-8; Dojindo, Tokyo, Japan). Cells were transfected with short interfering RNAs (siRNAs) siControl or siCBR1. Cells were incubated at a density of 5×10^3/well in 96-well culture plates (Costar, Cambridge, MA, USA) at 37 °C for 3 days, and every 24 h, each well of the cultured cells was incubated with 10 μl of Cell Counting kit-8 solution for 2 h at 37 °C. The absorbance was measured at 450 nm using an enzyme-linked immunosorbent assay reader (Molecular Devices, San Jose, CA, USA). The results are presented as percentages, relative to control cells.

Quantitative real-time PCR analysis

Total RNA was extracted from the indicated cell lines using an RNeasy mini kit (Qiagen, Hilden, Germany) according to the manufacturer's instructions. The RNA concentration was quantified using a NanoDrop spectrophotometer. Total RNA (500 ng) was reverse transcribed to cDNA using a qPCRBIO cDNA Synthesis kit (PCRBIOSYSTEMS. London, UK). The resulting cDNA was assayed by using quantitative real-time quantitative RT-PCR with 2× qPCRBIO SyberGreen Mix (PCRBIOSYSTEMS). Real-time RT-PCR was performed using the Step One Plus real-time RT-PCR system with a 96-well block module (Applied Biosystems, Foster City, CA, USA). The CBR1 primers were as follows: sense 5′-CAGAGACCCCTGTGTACTTG-3′; antisense 5′-CAACTCAGGACAAGG TACAAAATG-3′. The vascular endothelial growth factor A (VEGF-A) primers were as follows: sense 5′-CTACCTCCACCATGC CAAGT-3′; antisense 5′-CACACAGGATGGCTTGAAGA-3′. The VEGF-B primers were as follows: sense 5′-CCCTTGACTGTGGAGCTCAT-3′; antisense 5′-ACATCTCCCCCAGCT GACT-3′. The VEGF-C primers were as follows: sense 5′-

AGAGAACAGGCCAACCTCAA-3′; antisense 5′-GTTTGTCGCGACTCCAAACT-3′. The VEGF-D primers were as follows: sense 5′-AGGTTTGCGGCAACTTTCTA-3′; antisense 5′-ATCGGAACACGTTCACACAA-3′. Cycling conditions were 95 °C for 10 minutes, followed by 40 cycles of 95 °C for 15 seconds and 60 °C for 60 seconds. Relative amounts of mRNA were calculated from the threshold cycle number using expression of *GAPDH* (glyceraldehyde-3-phosphate dehydrogenase) as an endogenous control. All experiments were performed in triplicate and the values were averaged.

Nuclear/Cytosol Fractionation

After transfection, all cells were rinsed with ice-cold phosphate-buffered saline (PBS) and harvested using a cell scraper, followed by centrifugation. The cell pellets were used for nuclear/cytosol fractionation. The nuclear/cytosol extraction was prepared using an NE-PER Nuclear Cytoplasmic Extraction Reagent kit (Pierce, Rockford, IL, USA) according to the manufacturer’s instruction.
